# Clinical correlation of p16 expression with lymphatic invasion and epithelial-mesenchymal transition (EMT) in oropharyngeal carcinomas

**DOI:** 10.12688/f1000research.160134.2

**Published:** 2025-10-14

**Authors:** Vaishak Jawahar, Saraswathy Sreeram, Jyoti Kini, Sourjya Banerjee, Athiyamaan M S, Johan Sunny, Abhishek Krishna, Challapalli Srinivas, Dilson Lobo, Paul Simon, Bharat Sai

**Affiliations:** 1 Department of Radiation Oncology, Kasturba Medical College Mangalore , Manipal Academy of Higher Education, Karnataka, Manipal, India; 2 Department of Pathology, Kasturba Medical College Mangalore, Manipal Academy of Higher Education, Karnataka, Manipal, India

**Keywords:** HPV, oropharyngeal carcinoma, e-cadherin, vimentin, epithelial-mesenchymal transition, podoplanin, prognosis, survival.

## Abstract

**Background/Aim:**

To examine the clinical correlation of p16 expression with Epithelial–Mesenchymal Transition (EMT) markers and lymphatic invasion in OPSCC patients in terms of clinical status at presentation, subsequent progression, and survival.

**Methods:**

Tissue blocks of biopsy-proven Oropharyngeal Squamous Cell Carcinoma were subjected to Immunohistochemistry (IHC) for evaluating the expression of p16, e-cadherin, vimentin and podoplanin. This expression pattern was correlated with the demographic details, treatment response and survival patterns.

**Results:**

60 patients were finally available for evaluation in this study. Prevalence of HPV infection in our study was found to be 11.7%. E-cadherin expression was found in all HPV-associated patients whereas vimentin was not expressed in any of these. 71.4% patients had low Podoplanin expression and 85.7% had low lymphatic vessel count. Among the HPV- associated patients, 85.8% had T3-T4 stage, 100% had N2-N3 disease and 95% had stage IV disease. It was also found that p16-positive patients had significantly higher 1-year Overall survival (OS) (80%, p=0.045), higher 1-year Progression-free survival (PFS) (60%) and higher 1-year Locoregional recurrence-free survival (LRRFS) (75%) when compared to p16-negative patients.

**Conclusion:**

Prevalence of HPV infection was found to be similar to that of previous studies conducted in India. As previous literature suggests, the HPV-positive patients in our study presented with advanced nodal disease at presentation and thereby, an advanced overall stage. Further follow-up of these patients including their treatment details, determination of possible prognostic markers, and evaluation of their survival parameters can be done which can help in modifying the existing treatment modalities as HPV-associated OPSCC are known to have better prognosis according to literature.

## Introduction

Head and neck cancers (HNC) comprise a group of neoplasms involving the oral cavity, oropharynx, nasopharynx, hypopharynx, and larynx. It is typically associated with risk factors, such as excessive alcohol intake, tobacco exposure, or both. However, oropharyngeal tumors have been found to be strongly associated with human papillomavirus (HPV) infection, particularly HPV-16 and HPV-18 strains ( Johnson et al., 2020; Saxena et al., 2019). The main treatment approach for any pharyngeal malignancy is chemoradiation, predominantly owing to the higher functional morbidity observed following surgery in such patients. In general, HPV-associated HNC has been found to a better prognosis after treatment than non-HPV-associated HNC. Hence, multiple ongoing studies are exploring the scope of therapeutic dose de-escalation in the management of HPV-associated diseases ( Johnson et al., 2020).

The most prevalent histological subtype in HNC is the Squamous Cell Carcinoma variant known as head and neck squamous cell carcinoma (HNSCC) ( Saxena et al., 2019). It is the second-most prevalent form of cancer in India and comprises more than 30% of all the malignancies in India ( Sung et al., 2021). This increased incidence rate is because the majority of the Indian population has a lower socioeconomic status, increased trend in habits such as tobacco chewing and alcohol consumption, insufficient exposure to new diagnostic measures, and subsequent delayed reporting. Despite recent advances in surgery and radiotherapy techniques over the last few decades, there has not been a considerable increase in the survival rates of these patients ( Huber et al., 2011). Therefore, an extensive understanding of the prognostic factors in the interplay is of great importance for improving possible outcomes.

Although HPV-associated tumors of Oropharyngeal Squamous Cell carcinoma (OPSCC) often have advanced nodal disease at presentation, they respond better to treatment and, thereby, have a better prognosis. However, the exact molecular mechanisms underlying this phenomenon remain unknown ( Wakisaka et al., 2015). Tumor invasion generally occurs in two unique forms: single-cell invasion and collective cell invasion. Epithelial-Mesenchymal Transition (EMT) is a process in which tumor progression occurs via single-cell invasion, where epithelial tumor cells acquire mesenchymal cell properties, leading to dissemination and metastasis. The characteristic features of EMT include changes in epithelial characteristics, such as decreased expression of E-cadherin and β-catenin, and an increase in mesenchymal characteristics, such as increased expression of fibronectin, vimentin, and proteolytic enzymes, resulting in intercellular reorganization. EMT has been reported to be involved in tumor invasion and its distant spread ( Kalluri & Weinberg, 2010; Thiery, 2002). EMT has been observed in HNSCC and is generally associated with a poor prognosis ( Cappellesso et al., 2015; Jensen et al., 2015; Schrader et al., 2015; Frederick et al., 2007). The association of these EMT markers with the HPV status of patients and their prognosis in OPSCC has not been investigated in detail, despite limited studies including a smaller number of patients ( HATAkEYAMA et al., 2014; Wakisaka et al., 2015).

Podoplanin is a type-1 transmembrane glycoprotein, which in humans, are seen to be expressed in renal podocytes, placenta, skeletal muscle, heart, lungs, myofibroblasts of the salivary glands, breast, mesothelial cells, and osteoblasts ( Breiteneder-Geleff et al., 1999; Martín-Villar et al., 2005; Ordóñez, 2006). It is generally used as a specific surrogate marker for lymphangiogenesis, as it is exclusively expressed in the lymphatic vessels ( Breiteneder-Geleff et al., 1999).

Hence this study was conducted with an aim to establish a clinical correlation between p16 expression, lymphatic invasion and Epithelial Mesenchymal Transition (EMT) markers in OPSCC with the T-N status at presentation, response to treatment, survival and metastatic propensity.

## Methods

### Patient characteristics

The medical records of 141 patients who presented with the diagnosis of OPSCC at the Department of Radiation Oncology, KMC Attavar from the year 2017-2022 were initially evaluated for this longitudinal study after approval from the institutional ethics committee (Kasturba Medical College, Mangalore with IEC KMC MLR 12-2020/419 dated 24/12/2020). As the study involved retrospective analysis of the previously available specimens from treated cancer patients, the consent of the participants was waived off from the institutional ethics committee.

Of these, 81 patients who had either discontinued treatment, were lost to follow-up, or had inadequate/unavailability of histopathological specimens were excluded, and a final total of 60 patients were available for final evaluation. Staging of the patient was performed according to the 7th edition of the American Joint Committee on Cancer (AJCC)- TNM classification of malignant tumors, using the personal/medical data of the patients, including clinical notes and radiological imaging records, as the p16 expression assessment was performed after the treatment ( Edge et al., 2010). Tumor and lymph node status were divided into two categories (T1-T2 and T3-T4; N0-N1 and N2-N3, respectively). Other demographic details are also noted.

### Immunohistochemistry

The respective biopsy slides were reviewed with hematoxylin and eosin, and 3-micron thin sections were cut from the tumor blocks with the maximum tumor content and area of deepest invasion. Antibodies to p16, EMT markers (E-cadherin and Vimentin), and podoplanin were used for immunohistochemistry (IHC), and their expression patterns were assessed. Positive controls for all the above-mentioned markers were selected and used for the verification of each marker.

Formalin-fixed paraffin-embedded sections (3 µm) from tumor blocks enriched for deepest invasion were processed on an automated stainer using a polymer-HRP system. After deparaffinization and rehydration, endogenous peroxidase was blocked (3% H _2_O _2_, 10 min).

Primary antibodies and conditions (per marker):

p16-INK4 (clone: [MX007]; cat. no.: [MAD-00690QD-R-3]; supplier: [Master Diagnostics, DSS ImageTech Pvt Ltd.]) — dilution [ready to use])

E-cadherin (clone: [HECD-1]; cat. no.: [MAD-00761QD-R-3]; supplier: [Master Diagnostics, DSS ImageTech Pvt Ltd.]) — dilution [ready to use])

Vimentin (clone: [SP20]; cat. no.: [MAD-00326QD-R-3]; supplier: [Master Diagnostics, DSS ImageTech Pvt Ltd]) — dilution [ready to use])

Podoplanin (clone: [D2-40]; cat. no.: [MAD-00402QD-R-3]; supplier: [Master Diagnostics, DSS ImageTech Pvt Ltd]) — dilution [ready to use])

Retrieval: [buffer], pH [9.0], [preheat retrieval buffer for 5 min at high power 95 degree Celsius in microwave oven, with slides total 20 min, peroxidase blocking agent 10 min].

Primary incubation [40 min at RT]; polymer detection [Envision FLEX]; linker; secondary incubation (30 min RT); DAB chromogen; hematoxylin counterstain; graded ethanol dehydration and mount.

Controls: For each run, external positive controls were used (tonsil epithelium for p16; colonic mucosa for E-cadherin; fibroblasts for vimentin; lymphatic endothelium for podoplanin), along with on-slide internal controls when present. Negative controls replaced the primary antibody with isotype buffer.

Scoring and thresholds: p16 positivity required strong, diffuse nuclear and cytoplasmic staining (block-type pattern). E-cadherin, vimentin, and podoplanin were assessed in tumor at the invasive front. Two separate 20× fields containing ≥200 tumor cells in total were evaluated (with calibrated field area specified below). Positivity thresholds were pre-specified based on prior head-and-neck literature and our pilot runs: >20% tumor cells staining for E-cadherin or vimentin, and >10% for podoplanin. Podoplanin intensity/extent patterns were categorized (diffuse/focal × strong/moderate/weak) and dichotomized into high versus low/no expression as originally described.

For Lymphatic vessel count (LVC), On D2-40 sections, the highest-density non-overlapping hotspots were selected at scanning magnification; lymphatic profiles were counted at 100× in each hotspot using the calibrated 100× field area. In keeping with our original definition, LVC > 3 was considered high lymphangiogenesis. Lymphatic vessel density was assessed in sections stained with anti-PDPN. Using low power, the area with the largest density was chosen, and counting was done using 100× magnification. Lymphatic Vessel Count (LVC) >3 was considered high lymphangiogenesis in the tumor ( Wakisaka et al., 2015).

**Figure 1.**
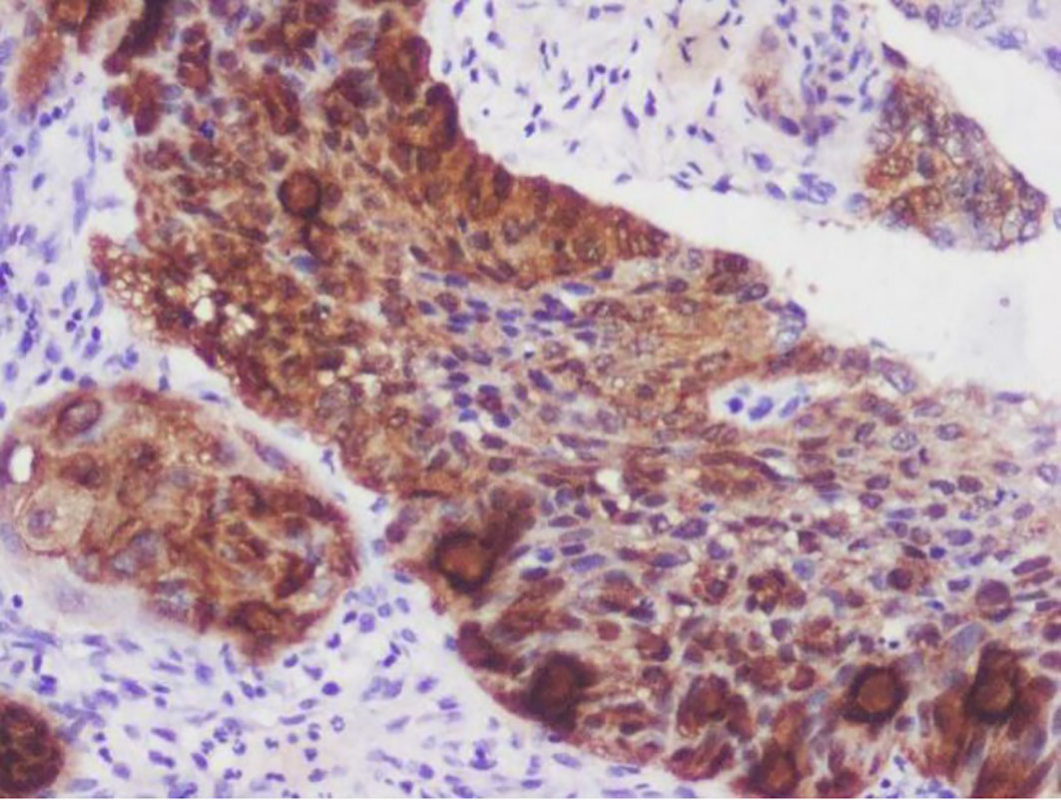
Expression of p16.

**Figure 2.**
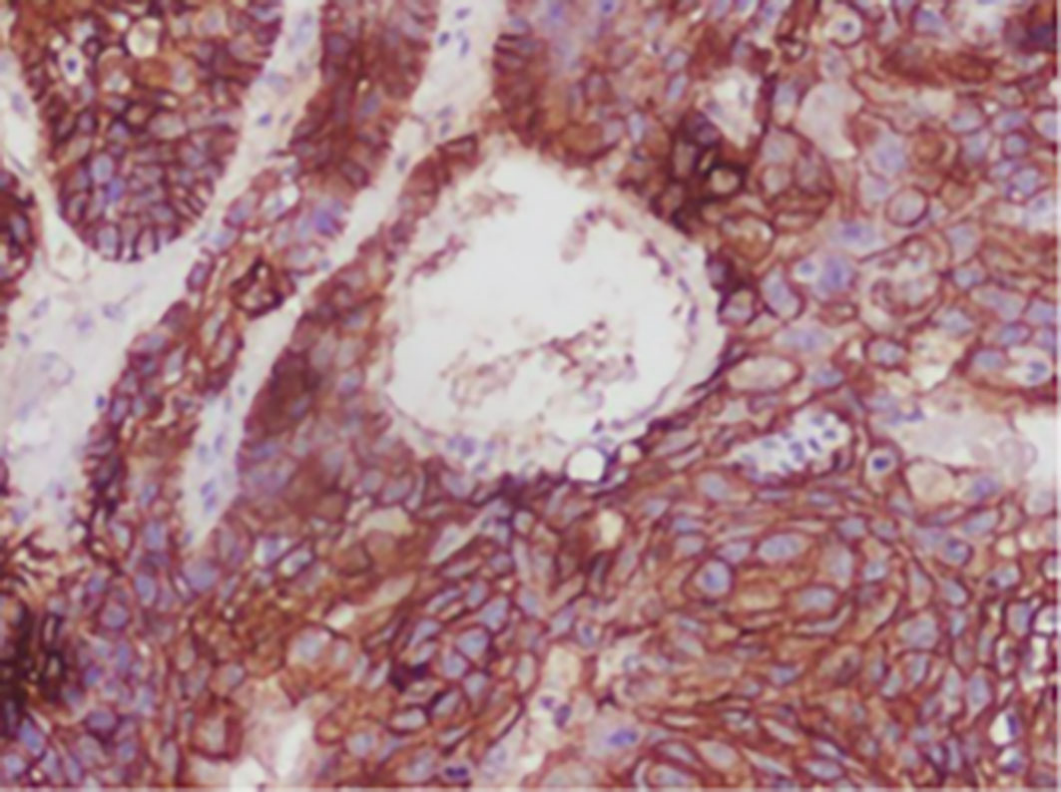
Expression of E-CADHERIN.

**Figure 3.**
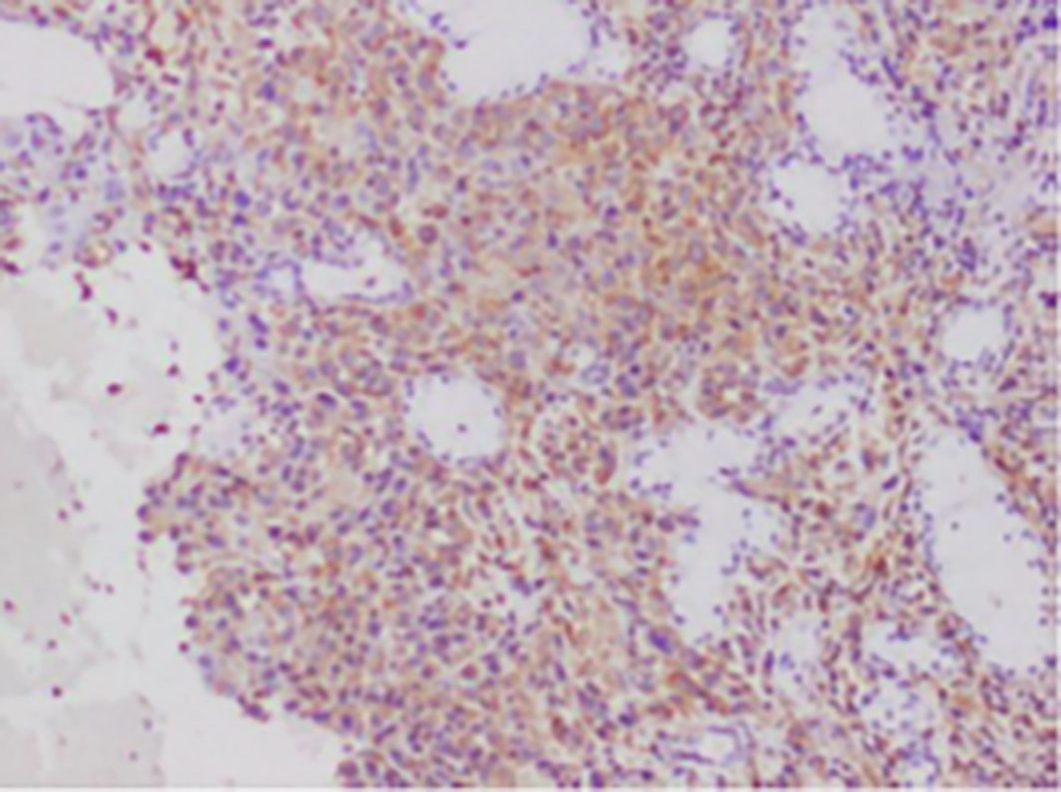
Expression of VIMENTIN.

**Figure 4.**
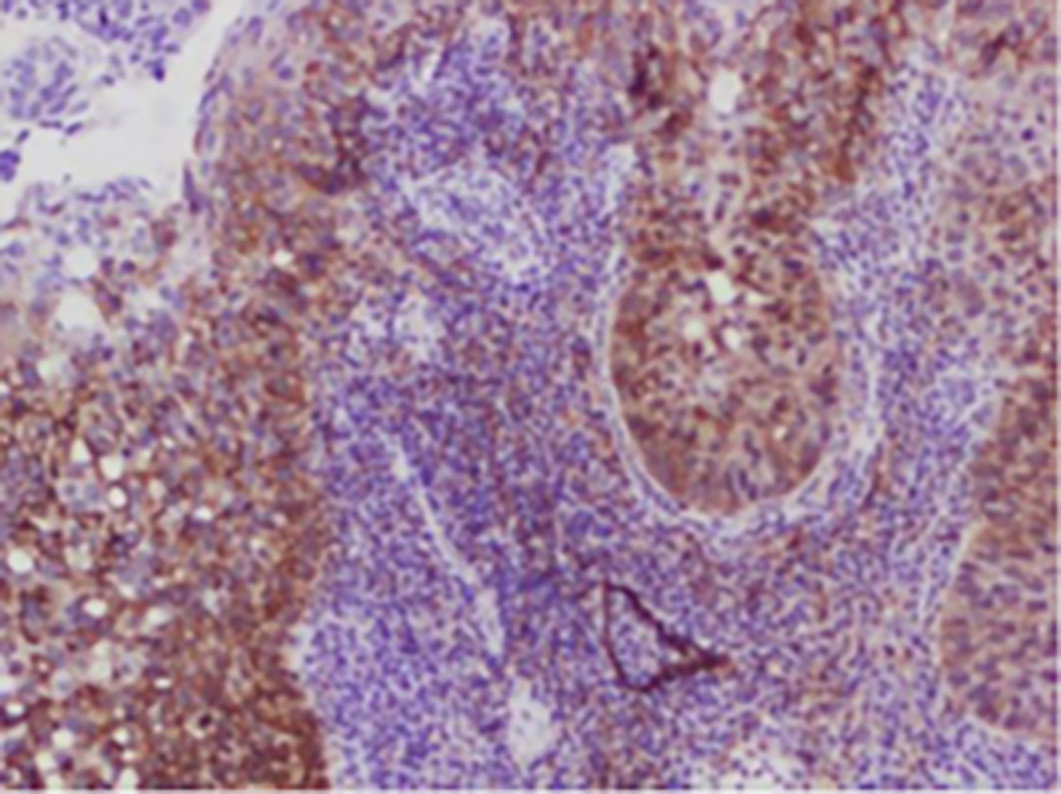
Expression of PODOPLANIN.

The IHC-stained slides were assessed separately by two pathologists. To avoid bias during assessment, they had no access to the patients’ clinical data. During the occurrence of inter-observer variation in assessment, specific slides were made to be viewed together, and their consensus opinion was taken into consideration ( Figures 1- 4).

### Treatment response and survival parameters

The treatment response and current disease status of the patients were assessed at 3, 6, and 12 months after the completion of treatment. Treatment response evaluation was based on clinical examination, which was confirmed using radiological imaging whenever there was suspicion. Retrospective data were also analyzed in the same way. Patients were supposed to have had a complete response to treatment when there was no clinical evidence of disease during follow-up. Partial response was defined as a reduction in the size of the primary tumor or node during follow-up. Any increase in the size of the primary/node or appearance of any new primary/node/metastatic lesion elsewhere was defined as a progressive disease. If there was no change in the size of the disease clinically, it was defined as stable disease. Survival parameters including Overall Survival (OS), Progression-Free Survival (PFS), and Locoregional Recurrence-Free Survival (LRRFS) were calculated. Patients who failed to return for follow-up visits were contacted telephonically to assess their disease status. A patient was considered to be lost to follow-up if they failed to respond even after a second phone call.

### Statistical analysis

Data was entered in Microsoft Excel and Statistical Analysis was done with the help of SPSS version 22 (IBM Corp. Released, 2013. IBM SPSS Statistics for Windows, Version 22.0. Armonk, NY: IBM Corp.).

Descriptive analysis was used to indicate the distribution of the quantitative variables in terms of Mean, Standard Deviation (S.D), median, and interquartile range (IQR). Qualitative variables are defined as percentages by category. The association between p16 expression, EMT, and lymphatic invasion with T status, N status, response to treatment, PFS, OS, and LRRF was calculated using the chi-square test.

Survival analysis was performed, and predictors of survival were identified. The Kaplan Meier graph was used to report the median survival time of patients with oropharyngeal carcinoma. Statistical significance was set at P < 0.05.

## Results

### Demographic data

The mean age of the 60 patients included in this study was found to be 58.27±10.09 and the median age was 60. The male: female ratio was 9:1 ( Table 1). The commonest subsite among the parts of the oropharynx involved in this study was found to be the tonsils (48.3%). Histopathological examination revealed that majority of the patients (60%) had moderately differentiated squamous cell carcinoma (MDSCC), followed by well-differentiated squamous cell carcinoma (WDSCC) (30%), and poorly differentiated squamous cell carcinoma (PDSCC) (10%). 83.4% patients had advanced T stage (T3-T4) and 78.4% patients had advanced N stage (N2-N3) disease. 46 out of 60 patients (76.7%) had Stage IV disease ( Table 2).

**Table 1.** Demographic data 1.

Age (in years)	Number of patients	Percentage (%)
<60	29	48.3
>60	31	51.7
**Total**	60	100.0
**Mean ± SD**	58.27±10.09	
**Median**	60	
**Range**	47	

**Table 2.** Demographic data 2.

	Number of patients	Percentage (%)
**GENDER**		
Male	54	90
Female	6	10
**SUBSITE**		
Base of Tongue	22	36.7
Soft Palate	8	13.3
Tonsil	29	48.3
Uvula	1	1.7
**HISTOLOGICAL GRADE**		
Well-differentiated	18	30
Moderately differentiated	36	60
Poorly differentiated	6	10
**T STAGE**		
T1-T2	10	16.6
T3-T4	50	83.4
**N STAGE**		
N0-N1	13	21.6
N2-N3	47	78.4
**OVERALL STAGE**		
I	0	0
II	3	5
III	11	18.3
IV	46	76.7

### Prevalence of hpv infection

Seven of 60 OPSCC patients (11.7%) in this study had positive p16 expression and hence were deemed to be having HPV-related OPSCC ( Table 3).

**Table 3.** Prevalence of HPV.

p16 status	Number of patients	Percentage (%)
**Negative**	53	88.3
**Positive**	7	11.7
**Total**	60	100.0

### Immunohistochemical detection and correlations

42 patients (70%) were found to have e-cadherin expression in this study. Three patients (5.7%) were found to have vimentin expression. Only one patient had EMT positivity. Totally 22 out of 60 patients (36.7%) were found to have high podoplanin expression. In the present study, the median LVC was 3. 34 patients (56.7%) had low LVC.

85.8% of all p16 positive patients had moderate well-differentiated disease (p=0.616). All the patients (100%) who were p16 + (HPV-associated) were found to be having e-cadherin expression (p=0.065). None of the p16+ patients showed vimentin expression (p=0.518). Only the patient with EMT positivity was p16 negative (p=0.714) ( Table 4).

**Table 4.** Correlation of HPV status with other IHC-marker expressions.

IHC-Marker Expression	Total	HPV-Positive	HPV-Negative	p
**E-Cadherin **				
Positive	42 (70%)	7 (100%)	35 (66%)	0.065
Negative	18 (30%)	0 (0%)	18 (34%)	
**Vimentin**				
Positive	3 (5%)	0 (0%)	3 (5.7%)	0.518
Negative	57 (95%)	7 (100%)	50 (94.3%)	
**EMT**				
Positive	1 (1.9%)	0 (0%)	1 (1.9%)	0.714
Negative	59 (98.3%)	7 (100%)	52 (98.1%)	
**Podoplanin**				
Positive	22 (36.7%)	2 (28.6%)	20 (37.7%)	0.329
Negative	38 (63.3%)	5 (71.4%)	33 (62.3%)	
**LVC**				
High (≥3)	26 (43.3%)	3 (42.9%)	23 (43.4%)	0.978
Low (<3)	34 (56.7%)	4 (57.1%)	30 (56.6%)	

p16 + patients (71.4%) had higher podoplanin expression when compared to p16 – patients (62.3%) (p=0.329). 57.1% of p16 positive patients were found to have low LVC count. Six of 7 p16 positive patients had a higher T stage (T3-T4) (p=0.857). All patients who were p16 + were found to have a higher nodal stage of N2-N3 (p=0.139) and consequently, Stage IV disease (p=0.299) ( Table 4).

90.4% Of all patients with E-cadherin positivity had moderate-well differentiated SCC. (p=0.690) Of the 42 patients who were positive for e-cadherin, 35 patients (83.4%) had a higher T stage (T3-T4) (p=1), 33 patients (78.6%) had advanced nodal disease (N2-N3) (p=0.945) and 34 patient (81%) had Stage IV disease (p=0.091). All patients having vimentin expression had moderate-well differentiated SCC, higher T Stage (T3-T4), advanced nodal disease (N2-N3) and Stage III-IV disease. Only one patient had EMT positivity and was found to have WDSCC, higher T Stage (T3-T4), high nodal Stage (N2-N3) and Stage IV disease.

71.8% Of all patients with high staining, 71.8 podoplanin had moderately well-differentiated SCC, whereas 94.7% of patients with low staining for podoplanin had moderately well-differentiated SCC. 86.4% Of all patients with high staining for podoplanin had T3-T4 stage whereas 91.6% of the patients with low staining for podoplanin had T3-T4 stage. 91% of the patients who had high podoplanin expression were found to have higher Nodal Stage (N2-N3). 86.3% Of the patients who showed high podoplanin expression were found to have Stage IV disease, whereas 71% of patients who showed low podoplanin expression were found to have Stage IV disease ( Table 5).

**Table 5.** Correlation of IHC-Marker expressions with respective demographic data.

IHC-Marker Expression	Histological Grade				Tumour status			Nodal status			Stage			
	WDSCC	MDSCC	PDSCC	p	T1-T2	T3-T4	p	N0-N1	N2-N3	p	II	III	IV	p

**p16**														
Positive (n=7)	3 (42.9%)	3 (42.9%)	1 (14.2%)	0.616	1 (14.2%)	6 (85.8%)	0.857	0 (0%)	7 (100%)	0.139	0 (0%)	0 (0%)	7 (100%)	0.299
Negative (n=53)	15 (28.4%)	33 (62.2%)	5 (9.4%)		9 (16.9%)	44 (83.1%)		13 (24.5%)	40 (75.5%)		3 (5.6%)	11 (20.8%)	39 (73.6%)	
**E-cadherin **														
Positive (n=42)	14 (33.3%)	24 (57.1%)	4 (9.5%)	0.69	7 (16.6%)	35 (83.4%)	1	9 (21.4%)	33 (78.6%)	0.945	3 (7.1%)	5 (11.9%)	34 (81%)	0.091
Negative (n=18)	4 (22.2%)	12 (66.7%)	2 (11.1%)		3 (16.6%)	15 (83.4%)		4 (22%)	14 (78%)		0 (0%)	6 (33.3%)	12 (66.7%)	
**Vimentin**														
Positive (n=3)	1 (33.3%)	2 (66.7%)	0 (0%)	0.839	0 (0%)	3 (100%)	0.427	0 (0%)	3 (100%)	0.35	0 (0%)	1 (33.3%)	2 (66.7%)	0.746
Negative (n=57)	17 (29.8%)	34 (59.7%)	6 (10.5%)		10 (17.5%)	47 (82.5%)		13 (22.8%)	44 (77.2%)		3 (5.2%)	10 (17.6%)	44 (77.2%)	
**EMT**														
Positive (n=1)	1 (100%)	0 (0%)	0 (0%)	0.305	0 (0%)	1 (100%)	0.602	0 (0%)	1 (100%)	0.596	0 (0%)	0 (0%)	1 (100%)	0.856
Negative (n=59)	17 (28.8%)	36 (61%)	6 (10.2%)		10 (16.9%)	49 (83.1%)		13 (22%)	46 (78%)		3 (5.1%)	11 (18.6%)	45 (76.3%)	
**Podoplanin**														
Positive (n=22)	5 (22.8%)	13 (59%)	4 (18.2%)	0.23	3 (13.6%)	19 (86.4%)	0.632	2 (9%)	20 (91%)	0.072	1 (4.5%)	2 (9.2%)	19 (86.3%)	0.357
Negative (n=38)	13 (34.2%)	23 (60.5%)	2 (5.3%)		7 (18.4%)	31 (91.6%)		11 (28.9%)	27 (71.1%)		2 (5.3%)	9 (23.7%)	27 (71%)	
**LVC**														
High (n=26)	8 (30.8%)	15 (57.7%)	3 (11.5%)	0.923	3 (11.5%)	23 (88.5%)	0.351	5 (19.2%)	21 (80.8%)	0.688	1 (3.8%)	4 (15.4%)	21 (80.8%)	0.44
Low (n=34)	10 (29.4%)	21 (61.7%)	3 (8.9%)		7 (20.5%)	27 (79.5%)		8 (23.5%)	26 (76.5%)		2 (5.9%)	7 (20.6%)	25 (73.5%)	

Of% patients with low LVC had T3-T4 stage whereas 88.5% patients with high LVC had T3-T4 stage. 76.5% Of patients with low LVC had N2-N3 disease, whereas 80.8% of patients with high LVC had N2-N3 stage. Of% patients with low LVC had moderately well-differentiated SCC, whereas 88.5% of patients with high LVC had moderately well-differentiated SCC. 80.8% Of patients a high LVC had Stage III-IV disease. Of% patients who had low LVC had Stage III-IV disease ( Table 5).

### Survival analysis and correlations

Of the 60 patients included in this study, 11 received palliative treatment. These patients were excluded, and the remaining 49 were included in the survival analysis. Of these 49 patients, only 23 showed a complete response after treatment. Hence, only these patients were considered in the calculation of the LRRFS.

Median OS for p16 positives patients was 14 months (IQR: 3-30 months) and the median OS among p16 negative subjects was 10 months (IQR: 5.75 – 12.25 months). 1-year OS rate was found to be significantly higher in p16-positive patients when compared to the of p16-negative patients (80% vs. 44.1%, p=0.045).

Median PFS for p16 positive patients was 6 months (IQR: 3-30 months) and the median PFS among p16 negative patients was 9.5 months (IQR: 3.75 – 12.25 months). 1-year PFS rate in p16-positive patients in our study was found to be 60% whereas, in p16-negative patients, it was found to be 31.8%.

**Figure 5.**
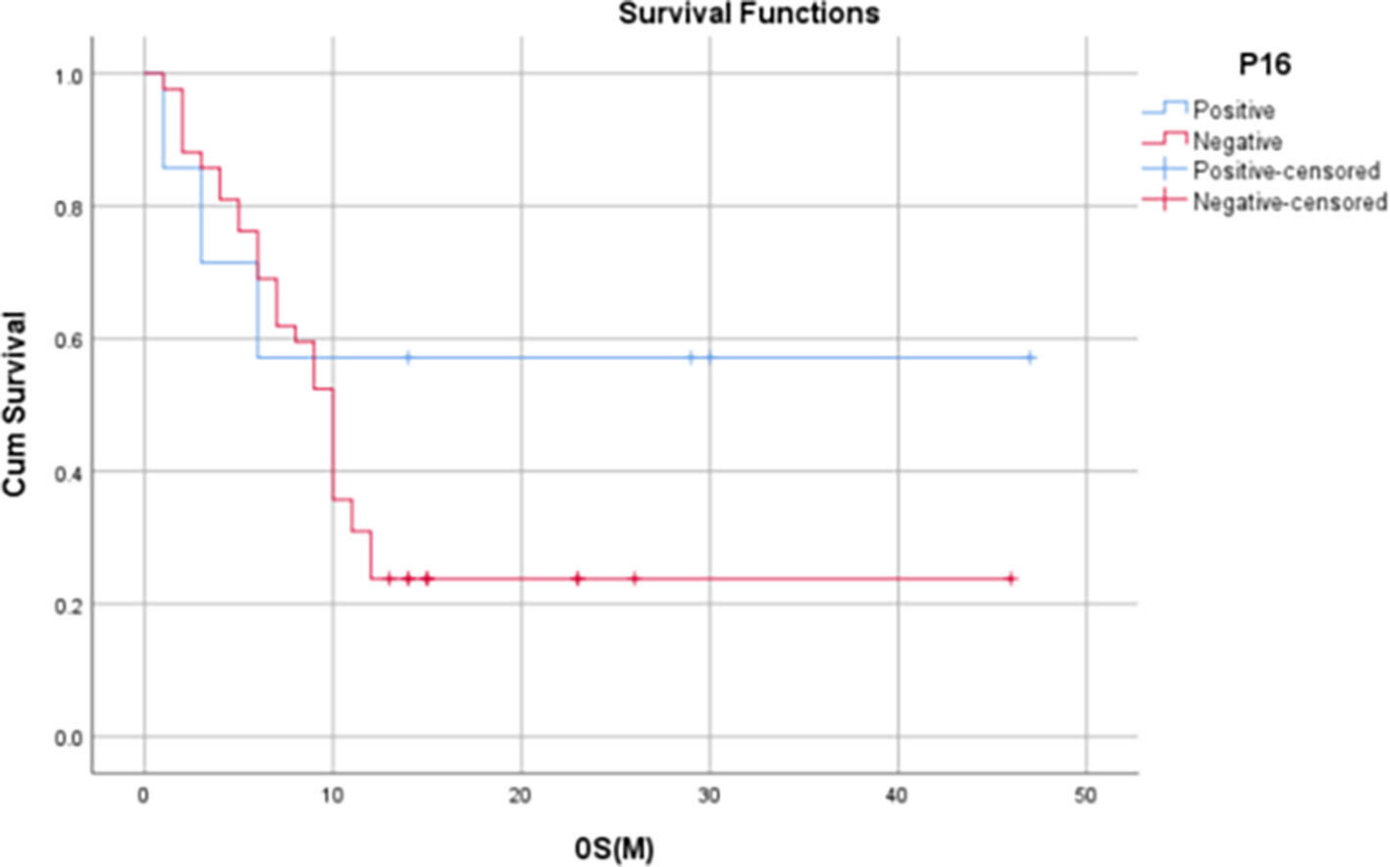
Kaplan Meier Curve 1 - Correlation of OS at 12 months with p16 status.

**Figure 6.**
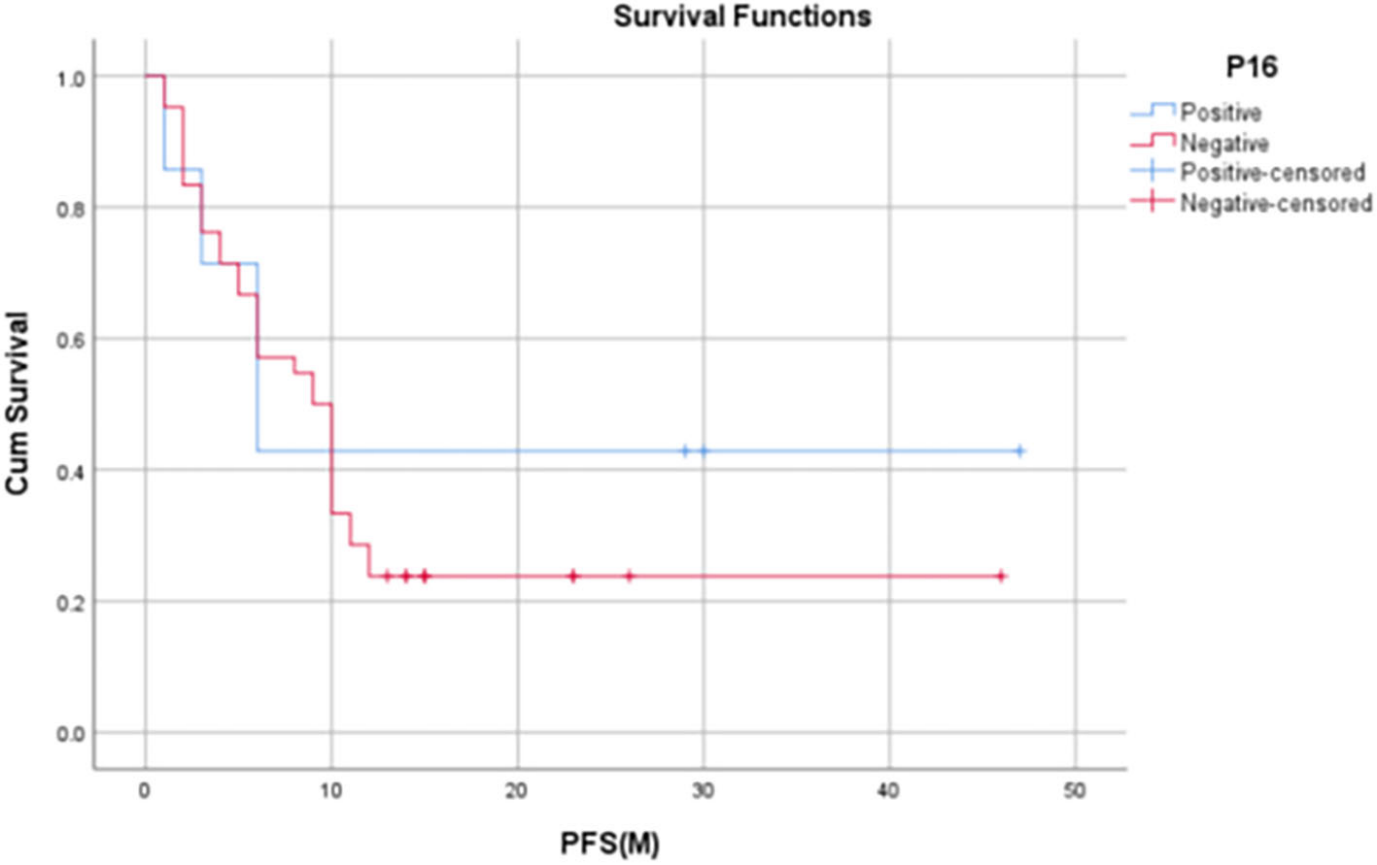
Kaplan Meyer Curve 2 - Comparison of PFS at 12 months with p16 status.

**Figure 7.**
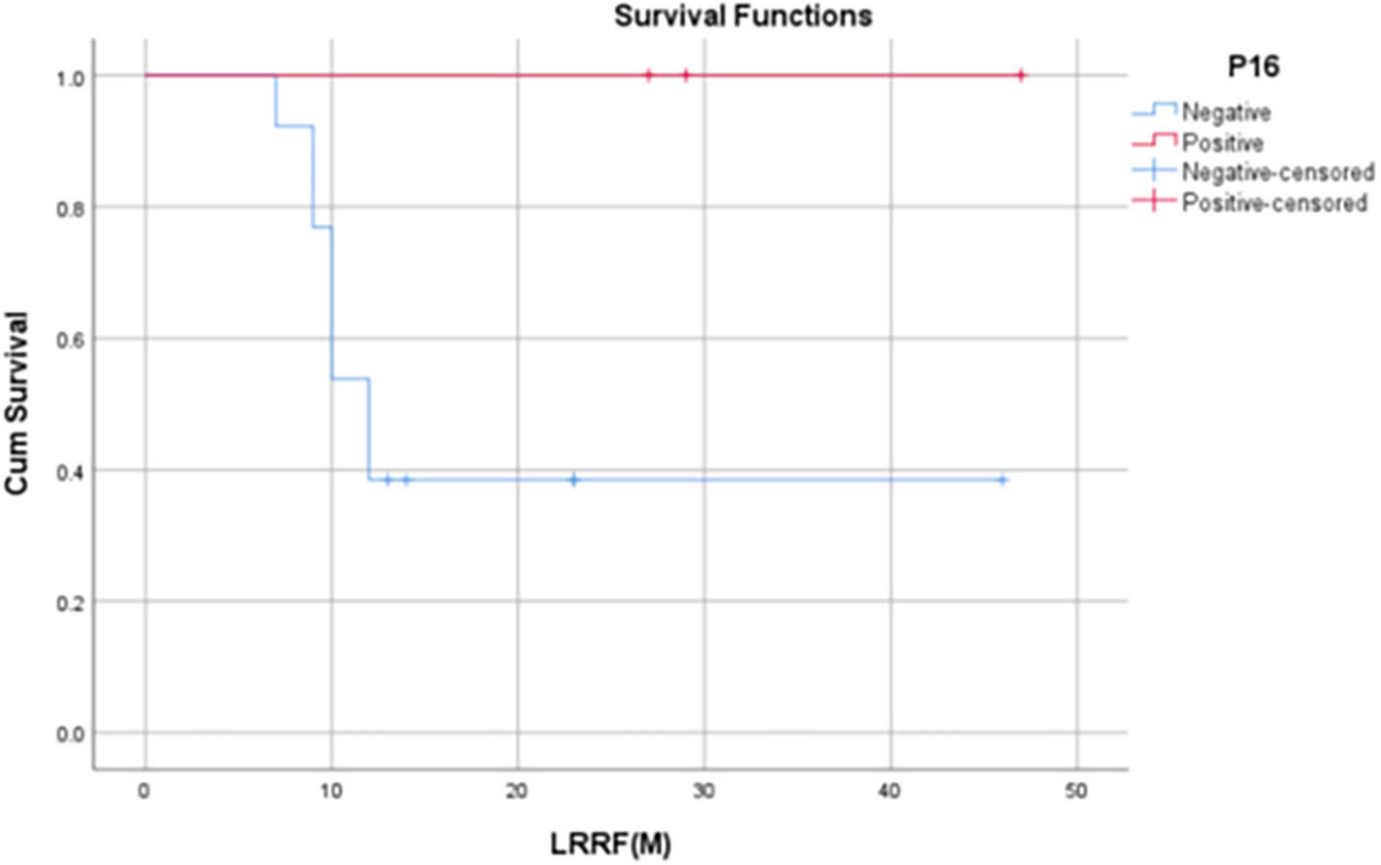
Kaplan Meier Curve 3 - Correlation of LRRFS at 12 months with p16 status.

The median LRRFS for p16 positive patients was 29 months (IQR: 17-29 months), and the median LRRFS among p16 negative patients was 12 months (IQR: 9.5– 18.5 months). 1-year LRRFS rate of p16-positive patients was found to be 75%, whereas it was 42% in p16-negative patients ( Table 6, Figures 5- 7).

**Table 6.** Correlation of HPV status with Survival parameters.

Survival parameter	HPV-Positive	HPV-Negative	Total	p
**OS at 1 year**				
Not Survived	1 (20%)	29 (65.9%)	49	0.045
Survived	4 (80%)	15 (34.1%)		
**PFS at 1 year**				
Not Survived	2 (40%)	30 (68.2%)	49	0.209
Survived	3 (60%)	14 (31.8%)		
**LRRF at 1 year**				
Not Survived	1 (25%)	11 (57.8%)	23	0.714
Survived	3 (75%)	8 (42.2%)		

## Discussion

According to previous studies, patients with HPV-associated OPSCC have been found to have aggressively growing tumors with advanced nodal disease at presentation, despite having a relatively better prognosis after primary treatment. Our study is in agreement with this finding.

A study conducted by Brittany J. Cline et al including 31702 patients revealed that the mean age at diagnosis was 60.3 years of which 77.1% were males and 22.9% were females ( Cline et al., 2020). Similarly, in the present study, the mean age at diagnosis was found to be 58.2. Ninety% of patients in our study were males, and the remaining 10% were females. According to a study by Tristan tham et al including 23297 OPSCC patients, the majority (96.4%) of the patients belonged to the subsite of tonsil. In the same study, it was found that the most common histological grade was poorly differentiated SCC (38.9%), followed by moderately differentiated SCC (31.1%) ( Tham et al., 2020). In our study, the most common site was found to the tonsil (48.3%). The most common histological grade in our study was moderately differentiated SCC (60%) followed by well-differentiated SCC (30%).

Compared to our Indian community, the prevalence of HPV association in OPSCC in the Western population was substantially higher. the current HPV prevalence in OPSCC in the US population is 70%, according to the CDC. The prevalence pattern of HPV infection related to OPSCC was as follows in a study by Catherine de Martel et al. to quantify the worldwide burden of cancer attributable to infections done in the year 2008 encompassing 12.7 million cases: North America, 56%; Northern and Western Europe-39%, Eastern Europe-38%, Southern Europe-17%, Australia-45%, Japan-52%, and rest of the globe-13% ( de Martel et al., 2012). Research conducted in India by Bahl et al. revealed 105 individuals with oropharyngeal cancer had a 22.8% HPV positive rate ( Bahl et al., 2014). Similar incidence rates of 20% and 15% were observed for Indian patients with oropharyngeal cancer by Murthy et al and Sannigrahi et al, respectively ( Murthy et al., 2016; Sannigrahi et al., 2016). In our investigation, it was shown that 11.7% of OPC had HPV infection. The slight variations in prevalence rates may be attributable to the chosen detection technique, which may include HPV DNA in situ hybridization, HPV DNA polymerase chain reaction, or immunohistochemistry (IHC) for the interpretation of p16 expression.

Wakisaka et al. conducted a study that included 53 OPC patients, in which 58.4% had strong E-cadherin expression, 64.1% had strong vimentin expression, 33.9% had EMT, and 62.2% had higher expression of podoplanin. In the present study, the median LVC was 3. Based on this value, 51% of the patients had low LVC count ( Wakisaka et al., 2015). In our study, the prevalence of strong E-cadherin expression was found to be 70%, and that of vimentin was found to be 5.7%. Only one patient (1.9%) had EMT positivity. 37.7% patients had High staining for podoplanin. In the present study, the median LVC was 3. According to this value, 56.6% of the patients had low LVC.

The influence of these parameters on the HPV status has been studied in multiple literature worldwide. However, few studies pertain to the Indian subpopulation. In a study by Wakisaka et al., 40.9% of all the HPV positive patients were found to have strong expression of E-cadherin, 90.9% were found to have vimentin expression, 60% were found to have EMT positivity, 63.6% were found to have low staining for podoplanin, and 63.6% were found to have low LVC. The same values for the HPV negative patients in their study were 70.9%, 45.2%, 19.4%, 9.7%, and 61.3%, respectively. In their study, 35.8% of the patients were found to have higher T stage (T3-4), 60.3% had higher N stage (N1-N3) and 75.4% had advanced stage disease (Stage III-IV).31.8% of HPV positive patients had T3-T4 stage, 77.2% had N1-N3 disease and 81.8% had Stage III-IV disease ( Wakisaka et al., 2015). In our study, out of all HPV positive patients, all the patients had E-cadherin expression (100%), none of the patients had vimentin expression, 100% had EMT positivity (only one patient had EMT positivity in the total sample), 71.4% were found to have low staining for podoplanin, and 85.7% had low LVC (Median LVC=3). The same values for HPV negative patients in our study were 66%, 100%, 0%, 62.3%, and 86.8%, respectively. Among the HPV positive patients present in our study, 85.8% had T3-T4 stage, 100% of the patients had N2-N3 disease and 95% had Stage IV disease. The low prevalence of vimentin is the primary reason why EMT was observed in only one patient. It is also evident that, although statistically insignificant, there is a definite correlation between positive E-cadherin expression and lower podoplanin staining with the association of HPV.

In a study by Hesham Mohammed et al. including 202 patients, out of all the E-cadherin positive patients, 53.1% had moderate well-differentiated disease, 42.3% had T3-T4 stage, 79% had N1-N3 stage and 82.1% had Stage III-IV disease. Out Of all the Vimentin positive patients in their study, 49.5% had moderate well-differentiated disease, 49.5% had T3-T4 stage, 79.1% had N1-N3 stage and 82.4% had stage III-IV disease ( Mohamed et al., 2020). In our study, among all the E-cadherin positive patients, 90.5% had moderate well-differentiated disease, 83.4% had T3-T4 stage, 78.6% had N2-N3 stage and 92.9% had stage III-IV disease. All vimentin-positive patients in our study had T3-T4 stage, N2-N3 stage and Stage IV disease. It is clear from this that Vimentin expression can be considered as a prognostic indicator for an advanced disease.

Various studies have shown that podoplanin expression corresponds to nodal progression, and thereby, to advanced disease ( Wakisaka et al., 2015). In our study, among the patients with higher Podoplanin expression, 81.8% had moderate well-differentiated disease, 86.4% had T3-T4 stage, 91% had N2-N3 disease and 86.3% had Stage IV disease. The same values for patients with low podoplanin expression were 94.7%, 91.6%, 71.1%, and 71%, respectively. These data are in good agreement with literature data showing that podoplanin can be considered a surrogate marker for lymphatic progression.

Posner et al studied (TAX 324) the association between HPV status and survival parameters in 264 patients with OPC. The respective tumor biopsies were subjected to PCR for HPV type 16 and assessed for correlation with demographic characteristics, OS, and PFS. Out Of the 264 patients, 42% had biopsy specimens which were evaluable of which 50% were found to be HPV-associated. 1 year-OS and PFS rates in HPV-related patients were significantly better than those in HPV-negative patients [93% vs. 69% (OS), 85% vs. 52% () respectively]. HPV-positive patients also had significantly better locoregional control than the HPV-negative patients (locoregional failure: 13% vs. 42%) ( Posner et al., 2011). Gypsyamber D’Souza et al. conducted a study that included 1356 patients, of which 517 were OPSCC and which 184 patients (35.6%) were found to be HPV-positive. HPV-positive patients had a significantly lower risk of mortality than HPV-negative patients. 3-year OS was significantly better in in HPV-positive patients (82%) when compared to HPV-negative patients (45%) ( D’Souza et al., 2016). In 2015, Arya et al. compared the HPV status of 3952 OPSCC patients with their respective OS rates. In this study, 62% patients were found to be HPV-positive. 2-year Overall Survival rate of HPV-positive patients was significantly higher compared to that of HPV-negative patients (93.1% vs. 77.8%) ( Amini et al., 2016). In our study, it was found that p16-positive patients had significantly higher 1-year OS rates when compared to that of p16-negative patients (80% vs. 44.1%, p = 0.045). Although not statistically significant, the 1-year PFS and 1-year LRRF survival rates were also found to be evidently improved among HPV-positive patients [60% vs 31.8% (PFS), 75% vs 42.2% (LRRFS)].

The small number of p16-positive cases limits power for subgroup analyses. Although p16 IHC is an accepted surrogate of transcriptionally active HPV, it can occasionally misclassify HPV-negative tumors as positive; confirmatory HPV testing (DNA/RNA PCR or ISH) was not performed and should be considered in future work.

Based on the results of our study, we concluded that HPV-positive status in OPSCC patients is significantly correlated with advanced nodal disease at presentation and improved clinical outcomes. Although not statistically significant, loss of podoplanin expression was found to have a clear correlation with nodal progression in HPV-positive status, similar to previous studies. In contrast to other studies, loss of E-cadherin, Vimentin expression and EMT positivity were not found to have any significant correlation with p16 expression. The improved survival pattern observed among HPV-positive patients was in agreement with previous studies. Hence, more intensive research is required to determine the exact molecular mechanisms involved in HPV-associated OPSCC. HPV prevalence can be evaluated using better diagnostic methods, such as HPV PCR or HPV ISH, to obtain more accurate results. Moreover, further studies need to focus on using E-cadherin, Vimentin and Podoplanin as prognostic markers for HPV-negative patients.

## Ethics and consent

approval from the institutional ethics committee (Kasturba Medical College, Mangalore with IEC KMC MLR 12-2020/419 dated 24/12/2020). As the study involved retrospective analysis of the previously available specimens from treated cancer patients, the consent of the participants was waived off from the institutional ethics committee.

## Data Availability

Figshare Repository: Underlying data for “Clinical correlation of p16 expression with lymphatic invasion and epithelial-mesenchymal transition (EMT) in oropharyngeal carcinomas”, https://doi.org/10.6084/m9.figshare.28099709.v1 ( Athiyamaan, 2024). The project contains the following underlying data:
• Book1 edited hpv.xlsx Data are available under the terms of the Creative Commons Attribution 4.0 International license (CC-BY 4.0).
