# Supplementary material for: Clinical correlation of p16 expression with lymphatic invasion and epithelial-mesenchymal transition (EMT) in oropharyngeal carcinomas
Source: F1000Res. 2025 Oct 14;14:121. Originally published 2025 Jan 22. [Version 2] doi: 10.12688/f1000research.160134.2 (PMC12553974; doi:10.12688/f1000research.160134.2)
Supplement: Peer review history

# Reviewer report: Reviewer response for version 2

**DOI:** 10.5256/f1000research.189525.r424052

**Published:** 2025-10-25

**Authors:** Agapiti H Chuwa

**Affiliations:** 1 University of Dar es Salaam, Mbeya, Tanzania

No further comments. The authors adequately addressed the issues observed in the first version.

Is the work clearly and accurately presented and does it cite the current literature?

Partly

If applicable, is the statistical analysis and its interpretation appropriate?

Partly

Are all the source data underlying the results available to ensure full reproducibility?

Yes

Is the study design appropriate and is the work technically sound?

Partly

Are the conclusions drawn adequately supported by the results?

Partly

Are sufficient details of methods and analysis provided to allow replication by others?

No

Reviewer Expertise:

Oncology, Gynecologic oncology, Reproductive medicine, Molecular physiology.

I confirm that I have read this submission and believe that I have an appropriate level of expertise to confirm that it is of an acceptable scientific standard.

# Reviewer report: Reviewer response for version 2

**DOI:** 10.5256/f1000research.189525.r424051

**Published:** 2025-10-15

**Authors:** Lanisha Sequeira

**Affiliations:** 1 Father Muller Medical College, Mangalore, India

The authors have addressed to all my queries.

Is the work clearly and accurately presented and does it cite the current literature?

Yes

If applicable, is the statistical analysis and its interpretation appropriate?

Yes

Are all the source data underlying the results available to ensure full reproducibility?

Yes

Is the study design appropriate and is the work technically sound?

Yes

Are the conclusions drawn adequately supported by the results?

Yes

Are sufficient details of methods and analysis provided to allow replication by others?

Yes

Reviewer Expertise:

Head and Neck Cancers, Brachytherapy, Medical Physics, Gynaecological Cancers

I confirm that I have read this submission and believe that I have an appropriate level of expertise to confirm that it is of an acceptable scientific standard.

# Reviewer report: Reviewer response for version 1

**DOI:** 10.5256/f1000research.175967.r411051

**Published:** 2025-09-11

**Authors:** Lanisha Sequeira

**Affiliations:** 1 Father Muller Medical College, Mangalore, India

Only one patient demonstrated EMT positivity, making it difficult to draw meaningful correlations. The authors can include a line in discussion.

 p16 IHC alone was used as a surrogate for HPV infection. Since p16 can sometimes yield false positives, combining IHC with molecular methods (e.g., PCR, ISH) would have strengthened the conclusions. This limitation should be clearly stated.

 Survival analysis is interesting, but the difference in PFS and LRRFS is not significant. This needs clearer explanation.

 Page 10 : The text “ *Ninety% of patients *Should be: *“Ninety percent”*

Is the work clearly and accurately presented and does it cite the current literature?

Yes

If applicable, is the statistical analysis and its interpretation appropriate?

Yes

Are all the source data underlying the results available to ensure full reproducibility?

Yes

Is the study design appropriate and is the work technically sound?

Yes

Are the conclusions drawn adequately supported by the results?

Yes

Are sufficient details of methods and analysis provided to allow replication by others?

Yes

Reviewer Expertise:

Head and Neck Cancers, Brachytherapy, Medical Physics, Gynaecological Cancers

I confirm that I have read this submission and believe that I have an appropriate level of expertise to confirm that it is of an acceptable scientific standard, however I have significant reservations, as outlined above.

# Reviewer report: Reviewer response for version 1

**DOI:** 10.5256/f1000research.175967.r408139

**Published:** 2025-09-10

**Authors:** Agapiti H Chuwa

**Affiliations:** 1 University of Dar es Salaam, Mbeya, Tanzania

Summary:

 The manuscript addresses an important clinical question using retrospective IHC data from 60 OPSCC patients at an Indian tertiary center. It correlates p16 expression with EMT and lymphangiogenesis markers, staging, and survival, with only 7 patients (11.7%) p16-positive showing better one-year outcomes. While regionally relevant and ethically approved, the very small p16+ subgroup limits interpretation. Substantial methodological clarification and revised statistical analyses are required to make the article scientifically sound.

 Major Issues:

 1. Clarity and accuracy:

- The manuscript is generally readable and references several relevant works, though many are outdated. Issues include inconsistencies in reported numbers (e.g., conflicting LVC medians), use of the outdated AJCC 7th edition rather than the 8th edition (which redefined p16/HPV staging in OPSCC), and discussion sections that overstate findings given the very small p16+ subgroup.
- Justify use of AJCC 7th edition or, preferably, restage according to AJCC 8th.
- Specify LVC fields (state field size).
- Correct the contradiction regarding median LVC (Methods/Results: 3 vs. Discussion: 9) and ensure consistency across text, tables, and figures.

2. Study design:

- The retrospective IHC approach is acceptable for exploratory correlations, but the exclusion of 81/141 patients raises concerns about selection bias. Very small event counts (p16+ n=7) undermine statistical power, and some analyses (e.g., Chi-square) are inappropriate for such small expected cell counts. Insufficient IHC technical details further limit reproducibility.

3.  Methods:

- Although sharing the dataset on Figshare is commendable, the IHC protocol requires significant expansion. Missing details include: antibody clones, catalogue numbers, suppliers, dilutions, antigen retrieval (buffer, pH, heating), justification of scoring thresholds (>20% for E-cadherin/vimentin, >10% for podoplanin), staining controls, and inter-observer agreement. The algorithm for LVC counting (number and size of fields) also requires clarification. While p16 IHC is an accepted surrogate for HPV, the absence of confirmatory HPV PCR or ISH should be acknowledged and emphasized as a limitation.

4. Analyses:

 Kaplan–Meier analysis is appropriate, but:

- Exclusion of 11 palliative patients must be justified.
- Chi-square tests with very small cell counts are invalid; Fisher’s exact test or an alternative approach should be used.
- No multivariable analysis (e.g., Cox regression) was performed to adjust for confounders; if not feasible, explain why.
- Censoring rules, dates of last follow-up, and numbers at risk should be specified.
- Report exact p-values and provide confidence intervals for small subgroups.

5. Data availability:

- The deposition of the dataset on Figshare is a strength, but replication of findings requires inclusion of the full IHC protocol as outlined above.

6. Conclusions:

- The association of p16 positivity with improved one-year OS is supported by the dataset, but the very small subgroup (n=7) and potential biases severely limit inference. Statements suggesting treatment de-intensification are premature and should be moderated.

Is the work clearly and accurately presented and does it cite the current literature?

Partly

If applicable, is the statistical analysis and its interpretation appropriate?

Partly

Are all the source data underlying the results available to ensure full reproducibility?

Yes

Is the study design appropriate and is the work technically sound?

Partly

Are the conclusions drawn adequately supported by the results?

Partly

Are sufficient details of methods and analysis provided to allow replication by others?

No

Reviewer Expertise:

Oncology, Gynecologic oncology, Reproductive medicine, Molecular physiology.

I confirm that I have read this submission and believe that I have an appropriate level of expertise to confirm that it is of an acceptable scientific standard, however I have significant reservations, as outlined above.
